# Finite element analysis of the performance of additively manufactured scaffolds for scapholunate ligament reconstruction

**DOI:** 10.1371/journal.pone.0256528

**Published:** 2021-11-19

**Authors:** Nataliya Perevoshchikova, Kevin M. Moerman, Bardiya Akhbari, Randy Bindra, Jayishni N. Maharaj, David G. Lloyd, Maria Gomez Cerezo, Amelia Carr, Cedryck Vaquette, David J. Saxby

**Affiliations:** 1 Griffith Centre of Biomedical and Rehabilitation Engineering (GCORE), Griffith University, Gold Coast, QLD, Australia; 2 Biomechanics Research Centre, National University of Ireland Galway, Galway, Ireland; 3 Center for Extreme Bionics at the Media Lab, Massachusetts Institute of Technology, Cambridge, MA, United States of America; 4 Center for Biomedical Engineering and School of Engineering, Brown University, Providence, Rhode Island, United States of America; 5 School of Dentistry, University of Queensland, Herston, QLD, Australia; 6 School of Medicine, Griffith University, Gold Coast, QLD, Australia; University of Vigo, SPAIN

## Abstract

Rupture of the scapholunate interosseous ligament can cause the dissociation of scaphoid and lunate bones, resulting in impaired wrist function. Current treatments (e.g., tendon-based surgical reconstruction, screw-based fixation, fusion, or carpectomy) may restore wrist stability, but do not address regeneration of the ruptured ligament, and may result in wrist functional limitations and osteoarthritis. Recently a novel multiphasic bone-ligament-bone scaffold was proposed, which aims to reconstruct the ruptured ligament, and which can be 3D-printed using medical-grade polycaprolactone. This scaffold is composed of a central ligament-scaffold section and features a bone attachment terminal at either end. Since the ligament-scaffold is the primary load bearing structure during physiological wrist motion, its geometry, mechanical properties, and the surgical placement of the scaffold are critical for performance optimisation. This study presents a patient-specific computational biomechanical evaluation of the effect of scaffold length, and positioning of the bone attachment sites. Through segmentation and image processing of medical image data for natural wrist motion, detailed 3D geometries as well as patient-specific physiological wrist motion could be derived. This data formed the input for detailed finite element analysis, enabling computational of scaffold stress and strain distributions, which are key predictors of scaffold structural integrity. The computational analysis demonstrated that longer scaffolds present reduced peak scaffold stresses and a more homogeneous stress state compared to shorter scaffolds. Furthermore, it was found that scaffolds attached at proximal sites experience lower stresses than those attached at distal sites. However, scaffold length, rather than bone terminal location, most strongly influences peak stress. For each scaffold terminal placement configuration, a basic metric was computed indicative of bone fracture risk. This metric was the minimum distance from the bone surface to the internal scaffold bone terminal. Analysis of this minimum bone thickness data confirmed further optimisation of terminal locations is warranted.

## Introduction

**Fig 1:**
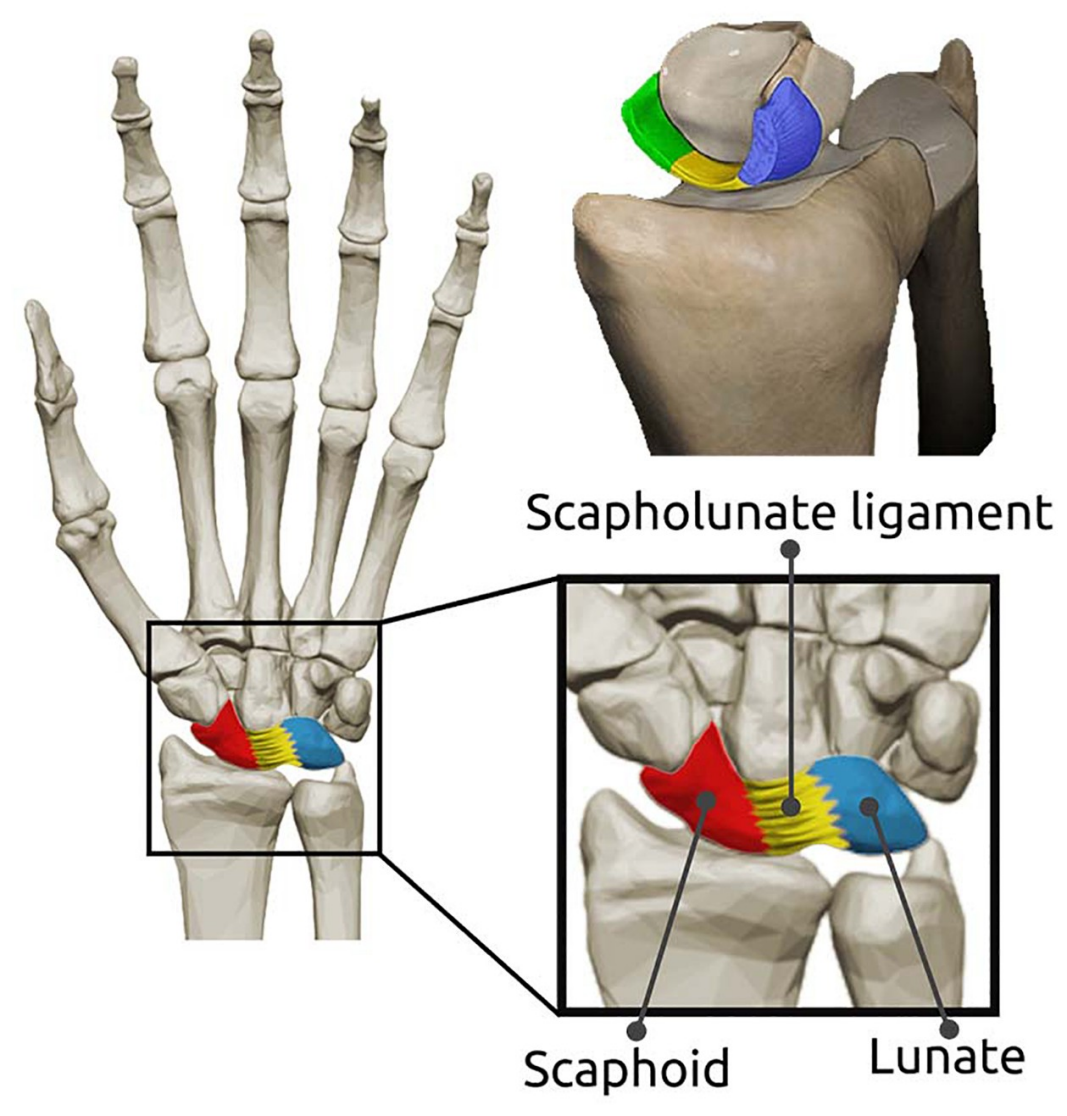
Wrist anatomy. Within the carpal bones of the wrist are the scaphoid and lunate bones. The scaphoid and lunate are held together by a the interosseous scapholunate ligament. A three-dimensional rendering of the scapholunate interosseous ligament is presented (top right). Removing the scaphoid reveals the dorsal (blue), proximal (yellow), and volar (green) regions of the the interosseous scapholunate ligament.

The human wrist is a complex joint that includes 8 small carpal bones arranged in two rows (Fig 1). The carpals are interconnected by intrinsic and extrinsic ligaments, and form at least 18 articulations. The scaphoid bone straddles both carpal rows and is bound to the lunate bone by the strong scapholunate interosseous ligament (SLIL). The SLIL is C-shaped and attached to the proximal edges of the scapholunate articulation (Fig 1, top right). The SLIL is comprised of three distinct anatomical regions, i.e. the dorsal, proximal, and volar region [1], which each presents distinct and anisotropic mechanical properties. The thickest and strongest of the three is the dorsal region, which is the primary stabiliser of the scapholunate joint [1–4], and is also mostly commonly injured during violent hyperextension of the wrist, such as during a fall [5].

Rupture of the SLIL can cause scapholunate (SL) dissociation, resulting in impaired wrist function and future onset of hand and wrist osteoarthritis. Studies of cadaveric wrists indicate only the dorsal SLIL region needs repair or reconstruction in order to restore normal carpal kinematics [6, 7]. Typically, complete SLIL rupture requires surgical intervention to restore wrist stability, with repair possible in early stages and reconstruction used for delayed presentation. Many different treatments for SLIL rupture have been suggested, with varying clinical results and poor reliability [8–15]. Usually, SLIL reconstruction involves a tendon-based approach [9–13] aiming to restore wrist stability using a tendon graft replacing the ruptured SLIL. However, tendon-based approaches present with inconsistent and often poor clinical outcomes. Indeed, 20-40% of patients go on to develop functional limitations and wrist osteoarthritis [16, 17]. Moreover, harvesting a portion of the flexor carpi radialis tendon as a graft (as is common) is problematic, as this muscle is important to dynamic wrist stability [18]. Furthermore, tendonous tissue may not accurately mimic the biomechanical properties of the SLIL, thus limiting its effectiveness for treating SLIL rupture [19].

**Fig 2:**
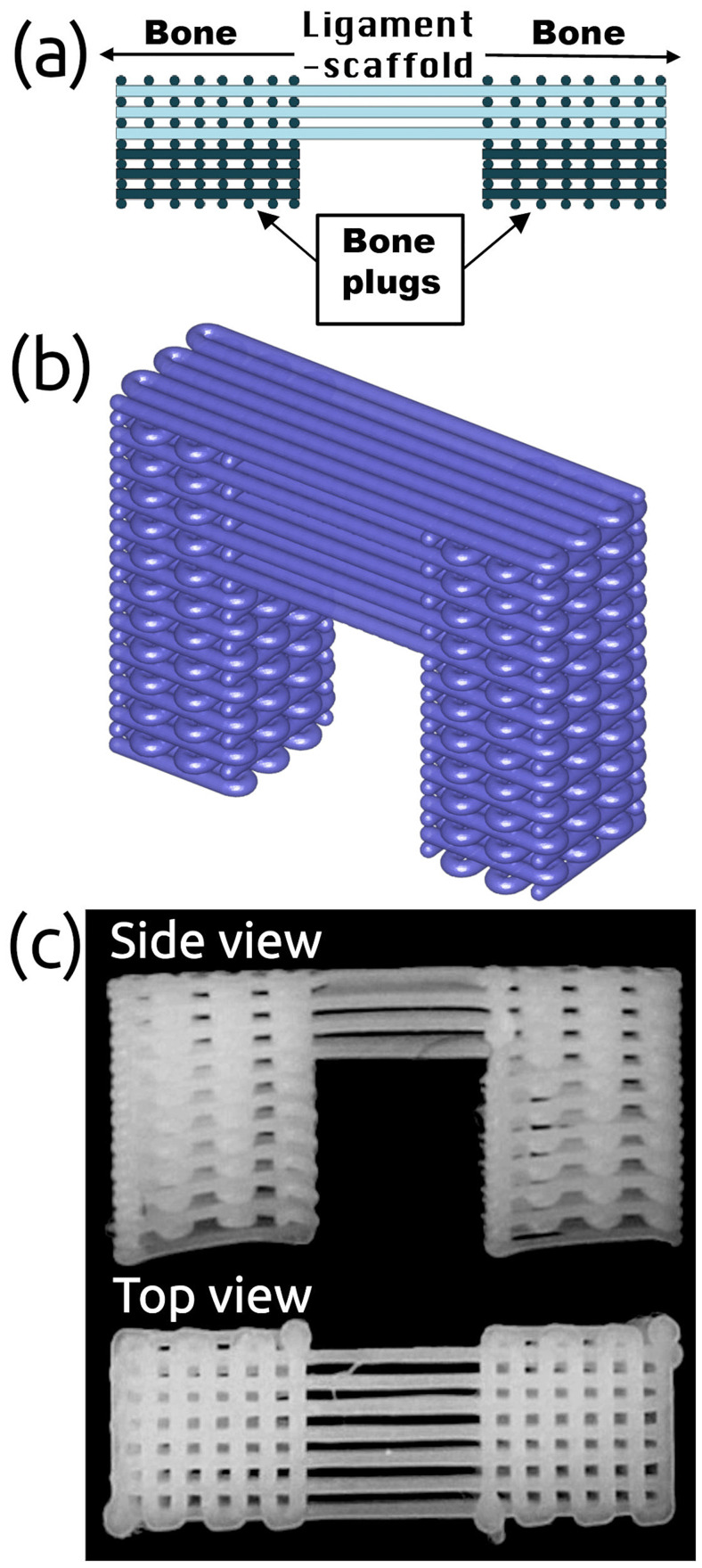
Design of multiphasic bone-ligament-bone scaffold. (a) A schematic 2D view and (b) 3D rendering of the scaffold design, and (c) an example fabricated scaffold.

Tissue engineering (TE) is a promising avenue for replacing injured anatomical structures of the hand [20, 21], especially given the limited availability of donor tissue. TE scaffolds produced from a variety of polymers [22, 23] should have interconnected pore structure and high porosity to ensure cellular penetration and adequate diffusion of nutrients to cells within the construct and to the extra-cellular matrix formed by these cells [20, 24, 25]. Additionally, they should have chemical, physical, biological, and adequate mechanical properties to perform as a replaceable frame [20, 26, 27]. Numerous conventional methods and advanced fabrication techniques have been introduced for scaffold manufacturing to replace tissue or organs employing solvent-casting particulate leaching [28] in combination with melt molding [29], gas foaming [30], and phase-separation [31]. More recently, 3D printing (digital light processing [32], fused deposition modeling [33] and robocasting [34]) have offered better control over the architecture and physical properties of the scaffold, thereby enabling the manufacturing of patient-specific constructs [35, 36]. Recently, a multiphasic bone-ligament-bone (BLB) scaffold [27] has been proposed to reconstruct the dorsal SLIL, and which can be 3D-printed using medical grade polycaprolactone (PCL). In this BLB scaffold, the ligament portion was represented by approximately parallel fibers terminating onto orthogonal plugs that interface with bone (Fig 2). Henceforth, the ligament portion will be referred to as the “ligament-scaffold”. *In-vivo* testing on small- and medium-sized animals demonstrated biocompatibility of the BLB, as evidenced by vascularisation and incorporation into the rabbit knee, as well as collagen deposition in the ligament-scaffold and bone formation within bone plugs [27, 37]. Despite these promising results from animal studies, use of BLB scaffold in the human wrist remains a non-trivial challenge due to the limited understanding of the physiological conditions of the human wrist, and the interaction between scaffold design and surgical installation. Indeed, sizing the scaffold for SLIL reconstruction is a careful balance between being small enough to fit in the small bones of the wrist, while being robust enough to avoid failure during physiological wrist motion.

This study presents a computational framework for the creation of personalised ligament-scaffold and associated surgical plan using finite element analysis (FEA). Given that the bone terminals are intended to be rigidly attached, or fused, to the bones, the ligament-scaffold portions of the BLB construct are subjected to the highest levels of deformation during physiological wrist motion. As such the focus of this study is a mechanical evaluation of the ligament-scaffold. After manufacturing the BLB scaffold using 3D-printing technique the computational framework encompassed: (1) extracting physiological wrist motions; (2) mechanical testing to determine material properties; (3) identification of material properties using inverse FEA; followed by (4) FEA of the scaffold under physiological motion; and (5) evaluation of the scaffold design. We hypothesized the ligament-scaffold will withstand (i.e., operate below failure stress) physiological wrist motion. Further, we hypothesized surgery positioning the ligament-scaffold distally (i.e., mimicking native SLIL attachment sites) will result in lower stresses during physiological motions compared to installation more proximal.

This is the first study to use detailed inverse finite element analysis to fit experimental data describing the hyperelastic properties of PCL material. Such large strain formulations are required for the current analysis and have not been presented before. By incorporating experimentally determined material parameters and realistic kinematic motion of the wrist, the FEA gives the rigorous assessment of stress within the scaffold. The use of accurate physiological wrist bone kinematics to assess scaffold mechanics through FEA has yet to be reported in the literature and may help future product developers establish physiological testing envelope for new devices at the wrist. In future, we will expand our admittedly limited sample (n = 1) to cadaveric wrist motion mobilized through robotic manipulation. This expanded data set will provide opportunity for more rigorous validation and sensitivity analysis of the FEA model to kinematic perturbation. Furthermore, this is the first open-source framework available for analysis of in-vivo wrist scaffold performance. This framework enables surgical planning as well as automated patient specific scaffold design.

## Methods

### Overview of the computational framework

**Fig 3:**
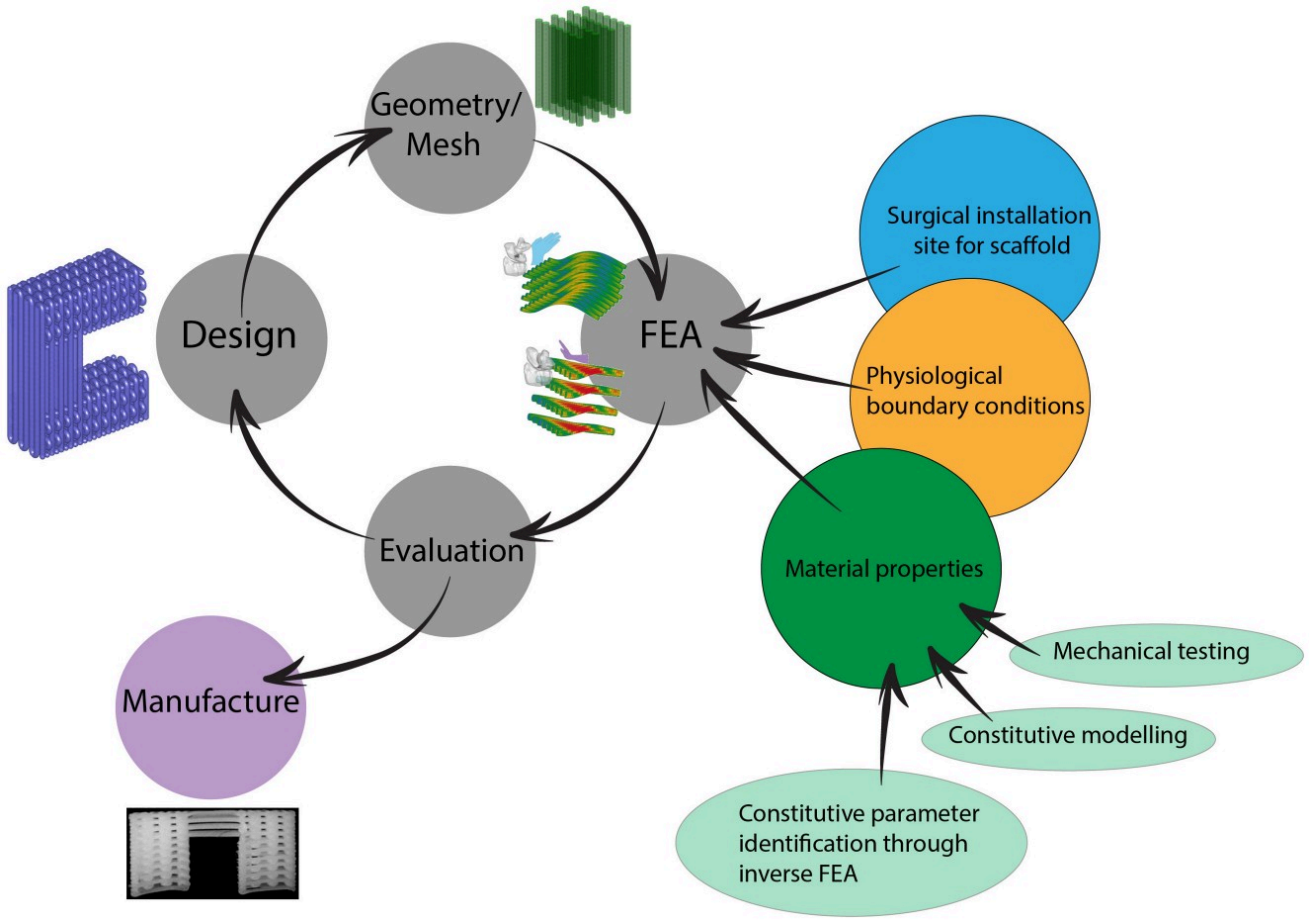
Schematic overview of the computational framework.

Fig 3 presents an overview of the computational framework. To start, the source design of the scaffold (denoted by the blue 3D picture in the Fig 3) is loaded and the geometry of the ligament-scaffold is meshed (green picture in the Fig 3). Next, several FEA models are created by (1) numerically placing the geometry according to surgical installation site for the scaffold (see Fig 5), (2) applying the physiological boundary conditions, and (3) assigning the material properties. The latter is derived from numerical fitting of the constitutive model to experimental mechanical data using inverse FEA. Finally, the FEA models are evaluated. If the evaluation is satisfactory, it can be sent to manufacturing. Otherwise, a re-design is necessary.

All data processing and visualization were performed using custom MATLAB (R2018b The Mathworks Inc., Natick, MA, USA) codes and the open source MATLAB toolbox GIBBON ([38], http://www.gibboncode.org/). FEA was implemented using the open source software FEBio (version 2.8.5, Musculoskeletal Research Laboratories, The University of Utah, USA, http://febio.org/ [39]).

### Scaffold design and manufacturing

The BLB scaffold used in this study was a modification of that developed by Lui and co-workers [27] and is made by 3D-printing of medical grade PCL (PC12 Corbion). The BLB scaffold design is depicted in Fig 2a and 2b in 2D, 3D, Fig 2c and an example fabricated scaffold, respectively. Overall, the scaffold consisted of two terminals orthogonal to the ligament-scaffold portion. The ligament-scaffold portion was composed of parallel PCL fibres mimicking native ligament structure. The terminals are intended to slot into wells drilled into the scaphoid and lunate, and are round cross-sections.

The scaffolds were 3D-printed using an 3D-Bioplotter Developer Series (EnvisionTEC GmbH), fitted with a 400 *μ*m Tecdia Arqué needle (ARQ-S-2030; Tecdia INC) attached to a high temperature head. The PCL was melted and extruded continuously to ensure strong cohesion between scaffold components and host environment which is essential for successful surgical installation and mechanical performance.

The BLB scaffold template (i.e., digital object) was loaded into the Perfactory Rapid Prototype (RP) software (EnvisionTEC, GmbH) wherein it was processed into 360 *μ*m slices (80% of printer needle diameter). The resulting file was transferred to the main software of the 3D-Bioplotter. Therein, the scaffold was assigned an infill comprising 0.7 mm fibre spacing (60% of porosity) and a 0–90° layer-to-layer rotation, resulting in 4 layers of fibres in the ligament compartment (total 28 fibres). The PCL material was placed in a stainless reservoir and heated at 110°C through a heated cartridge unit and extruded through a 400 *μ*m nozzle to deposit PCL fibres on a programmable stage at 2.5 mm/s.

### Surgical installation site for scaffold

**Fig 4:**
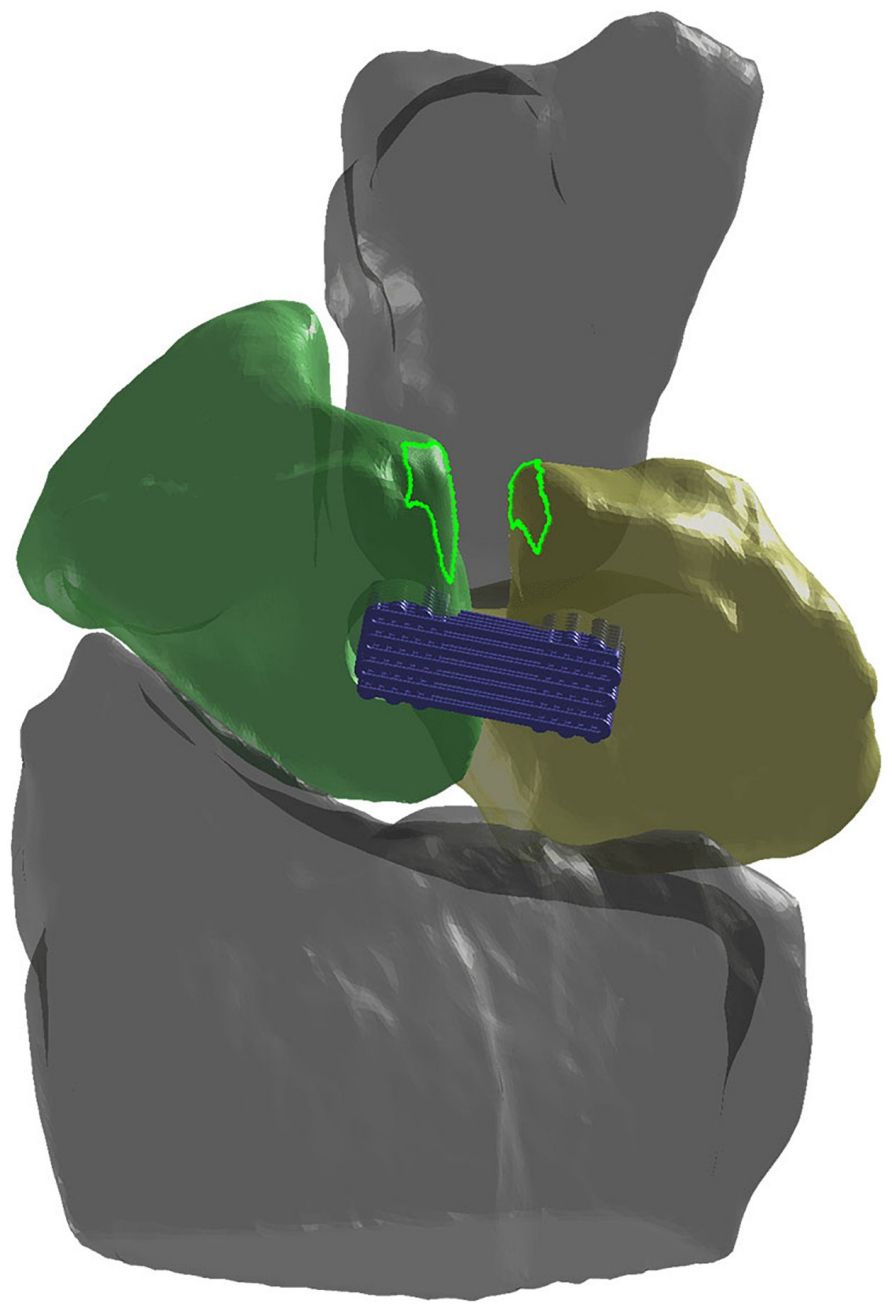
The 3D anatomical model created from computed tomography scan. The model consists of capitate (grey), scaphoid (green), lunate (yellow), and radius (grey) bones. Bone positions in dorsal view of anatomical model correspond to maximum wrist flexion. The attachment sites of the dorsal SLIL are depicted with green dotted lines on both scaphoid and lunate. The dorsal SLIL attachment sites are derived from MRI scan of intact native SLIL with the wrist at neutral position and projected onto the anatomical model created from computed tomography scan. The BLB scaffold is placed at a position proximal to the native attachment sites.

Fig 4 shows an anatomical model of a surgical site for scaffold installation and surrounding bony anatomy. Bright green lines indicate native SLIL attachment sites. In theory, to best approximate biomechanical function of the native SLIL scaffold terminals (“bone plugs”) should coincide with these attachment sites. In practice, surgical techniques employ more proximal attachment sites where there is greater bone volume to receive bone plugs and are deemed lower risk for bone fracture during surgical drilling. The location chosen for scaffold installation depicted in Fig 4 is a compromise between anatomical (and assumed biomechanical) fidelity and surgical risk.

**Fig 5:**
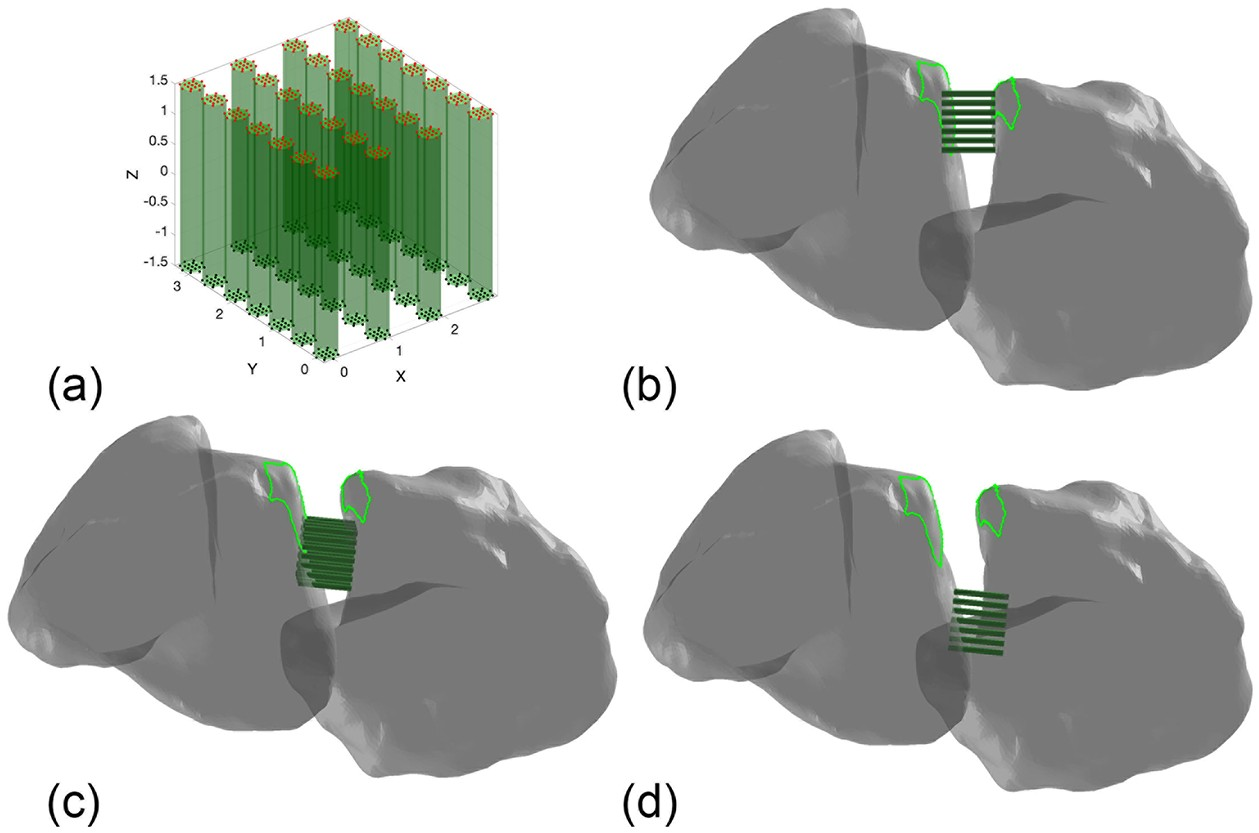
FEA models. (a) The 28 fibres represent the ligament-scaffold where red and black nodes are prescribed boundary conditions. Rigid bones demonstrate (b) distal, (c) intermediate and (d) proximal ligament-scaffold installation sites for FEA.

Three surgical installation sites for the ligament-scaffold portion (Fig 5a) of the BLB scaffold were assessed using FEA and shown in Fig 5b–5d. Due to the regional anatomy, each candidate site required a ligament-scaffold of different length. In total, twelve FEA models were developed based on different candidate installation sites and ligament-scaffold lengths, and subjected to the same motion boundary conditions from one human subject. In Fig 5b, the attachment sites of the dorsal SLIL depicted by green dotted lines on both scaphoid and lunate will be referred to as the distal surgical site. Typical surgical sites are more proximal to the native SLIL footprint in Fig 5 and 5d) and will be referred to as such. Finally, the surgical site between distal and proximal (Fig 5c) is proposed, and will be referred to as intermediate. Further, surgical installation of the scaffold should leave an intact wall of bone surrounding the plugs and countersink the ligament-scaffold beneath the surface to prevent impingement with other bones during wrist motion.

### Physiological boundary conditions

The kinematic boundary conditions (i.e., rotations and translations of scaphoid, lunate, and capitate) were derived from tri-planar videoradiography. The radius was mathematically fixed and kept static. Wrist motion was defined with reference to the capitate, which moves almost identically with the third metacarpal [40]. Using a similar approach to bi-planar videoradiography, tri-planar videoradiography combines a high-speed X-ray image sequences with 3D bone volumes and contours obtained from static CT scans to track 3D bone motion [41–44]. Motion data were collected from a single cadaveric specimen moved through two sets of flexion/extension rotations and ulnar/radial deviations.

For the tri-planar videoradiography, X-ray sources were oriented 120° relatively, with one parallel to the floor resting atop a trestle table. The cadaveric forearm was placed on the trestle table, and a wooden dowel used to manipulate the hand from outside the field-of-view. The hand was manipulated throughout flexion-extension and radial-ulnar deviation over one continuous 10 second data capture. The capitate, scaphoid, and lunate were tracked within the videoradiographic images, and were analyzed in a 2D-to-3D image registration software, Autoscoper (Brown University) [45]. Following registration, their 3D postures were calculated. Since surgical installation of the scaffold will be performed with the wrist flexed, wrist flexion was used as the reference frame and the path of flexion-extension-neutral-radial deviation-ulnar deviation was then used for FEA. Derived kinematic boundary conditions of scaphoid, lunate and capitate bones were used for FEA.

The experimental work to acquire the carpal database was approved by the Institutional Review Board (IRB) of Lifespan-Rhode Island Hospital, an AAHRPP accredited IRB.

### Mechanical testing

**Fig 6:**
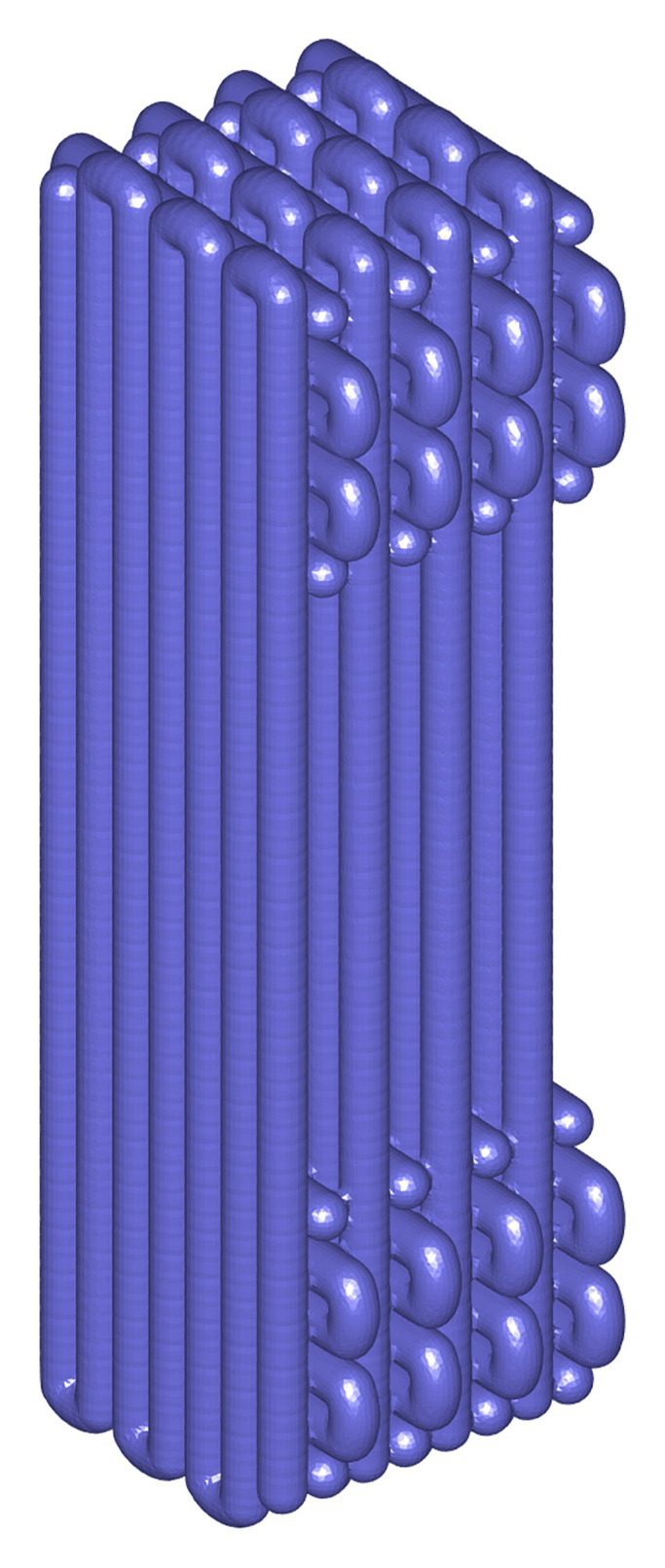
Geometry of rectangular sample. A 3D schematic of the rectangular sample used for uniaxial tensile and cyclic testings that consists of bone plugs and 10 mm length ligament-scaffold with 28 fibers and 350 *μ*m in diameter each.

To determine the PCL material behaviour, rectangular 3D-printed samples of PCL were used for uniaxial tensile and cyclic testings. Samples had an overall length of 15 mm, consisting of a ligament-scaffold portion 10 mm in length, and contained 28 fibers each of 350 *μ*m diameter (Fig 6), leading to a depth of 2.8 mm and a width of 5 mm. Seven monotonic and five cyclic tensile tests were performed. For these samples, the bone plugs were filled with epoxy resin (Selleys Araldite 5 Minute, Australia) to stiffen them before being inserted into pneumatic grips. This prevented compression and stress concentration at the borders between bone plugs and ligament-scaffold. Tensile testing was performed using a mechanical testing machine (5848 Microtester, Instron, USA) equiped with a 500 N load cell, tension was applied at a rate of 1/3 mm/s, and all tests were conducted at room temperature. The applied stretch and the Cauchy stress were calculated. The Cauchy stress is also called the true stress because it is a true measure of the force per unit area in the current, deformed, configuration. Cyclic testing was performed in phosphate buffer saline (PBS) at 37°C by applying 0.1 N preload. Samples were then subjected to 1000 continuous cycles of 10% strain at 0.5 Hz.

### Constitutive modelling

Constitutive equations were used to describe the mechanical behavior of the ligament-scaffold portion of BLB scaffold (Fig 5a) through specification of the dependence of force-displacement and force-time experimental data (see section above on Mechanical testing). The arithmetic average of force-displacement and force-time experimental curves were used for subsequent optimisation (described below). The ligament-scaffold solid material is modeled as homogeneous, isotropic, hyperelastic and viscoelastic.

Note that, although the 3D printed solid material is isotropic, a bulk 3D printed geometry may exhibit a different degree of anisotropy, indeed by printing a fibrous structure the bulk behaviour of the scaffold is transversely isotropic, similar to ligament and tendon tissue in-vivo.

The purely elastic behavior is represented by the following uncoupled hyperelastic strain energy density formulation Ψ:

$$\begin{array}{c} \Psi =\Psi_{dev}+\Psi_{vol} \end{array}$$ (1)

which features an additive decoupling of the deviatoric (shape changing) strain energy density Ψ_*dev*_, and the volumetric strain energy density Ψ_*vol*_. The former is here defined as:

$$\begin{array}{c} \Psi_{dev}\left({\tilde{\lambda }}_{1},{\tilde{\lambda }}_{2},{\tilde{\lambda }}_{3}\right)=\frac{c}{m^{2}}\sum_{i=1}^{3}({\tilde{\lambda }}_{i}^{m}+{\tilde{\lambda }}_{i}^{-m}-2) \end{array}$$ (2)

The variable *J* = λ_1_λ_2_λ_3_, with λ_*i*_ the principal stretches, represents the Jacobian or volume ratio. The variables ${\tilde{\lambda }}_{i}=J^{-}\frac{1}{3}\lambda_{i}$ are the deviatoric principal stretches. The parameter *c* is a shear-modulus-like material parameter with units of stress, and *m* is a parameter controlling the degree of non-linearity. Note that Eq 2 represents a second order Ogden formulation [46] (see also FEBio’s *Ogden* implementation), with the constraints *c* = *c*_1_ = *c*_2_, *m* = *m*_1_ = −*m*_2_ as motivated by [47]. Furthermore, note that if *J* ≈ 1 and *m* = 2 Eq 2 reduces to the well-known Mooney-Rivlin formulation. The volumetric strain energy density Ψ_*vol*_ in Eq 1 is given by:

$$\begin{array}{c} \Psi_{vol}\left(J\right)=\frac{\kappa }{2}\text{ln}{\left(J\right)}^{2} \end{array}$$ (3)

where *κ* represents the material bulk-modulus. The 3D printed PCL material was assumed to be nearly incompressible, as such the bulk modulus was here set at *κ* = 100 ⋅ *c*, which was found to be sufficient to keep volume changes below 1%. Using the strain energy density function Ψ the elastic second Piola-Kirchoff stress tensor **S** can be derived from:

$$\begin{array}{c} S=2\frac{\partial \Psi }{C}=2\frac{\partial \Psi_{dev}}{C}+pJC^{-1}=J^{-\frac{2}{3}}\text{Dev}[\tilde{S}]+\text{pJ}C^{-1} \end{array}$$ (4)

with $p=\frac{d\Psi_{\text{vol}}}{dJ}$ the hydrostatic pressure, **C** the right Cauchy green tensor, and $\tilde{S}$ an elastic stress contribution given by:

$$\begin{array}{c} \tilde{S}=2\frac{\partial \Psi_{dev}}{\partial \tilde{C}} \end{array}$$ (5)

where $\tilde{C}=J^{-\frac{2}{3}}C$ is the deviatoric right Cauchy-Green tensor. In Eq 4 use was made of the deviatoric operator in the Lagrangian description: $\text{Dev}[\tilde{S}]=\tilde{S}-\frac{1}{3}\left(\tilde{S}:C\right)C^{-1}$.

The above expressions are for purely elastic behaviour. To extend the formulation to capture the rate dependant viscoelastic behaviour of PCL the quasi-linear viscoelastic theory is used here (see also [48] and FEBio’s *Uncoupled viscoelastic* implementation). In this case the time dependant second Piola-Kirchoff stress tensor is now written:

$$\begin{array}{c} S\left(t\right)=J^{-2/3}\int_{-\infty }^{t}G\left(t-s\right)\frac{d(\text{Dev}[\tilde{S}])}{ds}ds+\text{pJ}C^{-1} \end{array}$$ (6)

where *G* represents the following discrete relaxation function:

$$\begin{array}{c} G\left(t\right)=1+\sum_{a=1}^{N}\gamma_{a}\text{exp}(\frac{t}{\tau_{a}}) \end{array}$$ (7)

The viscoelastic parameters *γ*_*a*_ have units of stress and represent proportional parameters, and *τ*_*a*_ have units of time and are relaxation time parameters. In this study a single relaxation term is used leading to *N* = 1, and the definition *γ*_1_ = *γ* and *τ*_1_ = *τ*. Note that the relaxation function *G* allows for the viscoelastic stresses to decay as a function of time (as per the chosen parameters *γ* and *τ*), such that once decayed, Eq 6 reduced to the purely elastic response defined by Eq 4.

### Constitutive parameter identification through inverse FEA

**Fig 7:**
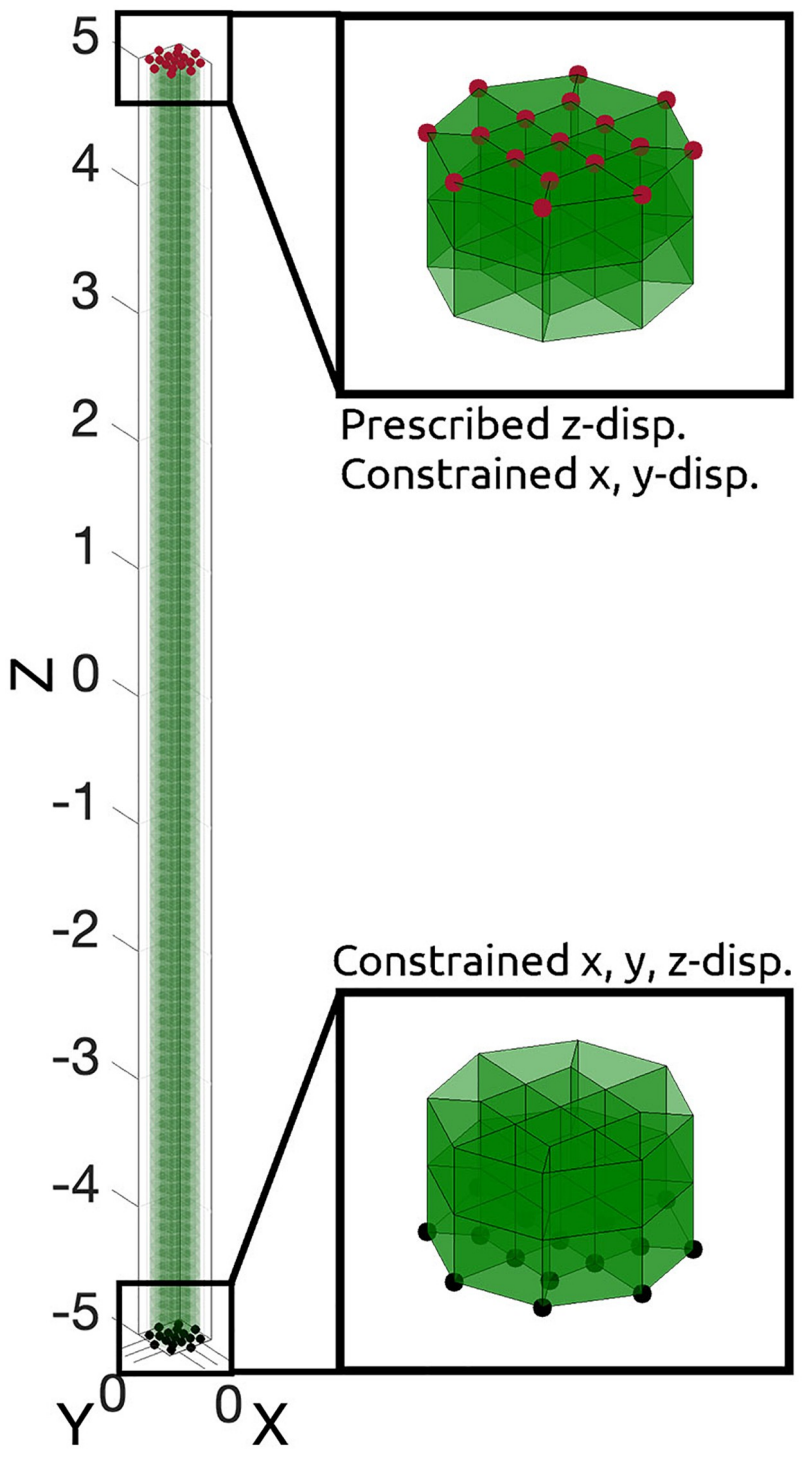
A single fibre. The hexahedral mesh of a single fibre (left) used for iFEA, and two close-ups for the top two and bottom two element layers. The top surface nodes (red) feature a prescribed z-displacement and a zero x, and y displacement constraint. The bottom surface nodes (black) are fully constrained from moving.

To identify PCL material parameters for the presented constitutive formulations, an iterative inverse FEA (iFEA) technique was used. Through iFEA a forward model is formulated which captures the experimental geometry and loading boundary conditions, and which features the appropriate constitutive formulations. The iFEA model is initiated with initial constitutive parameters and a difference between the experiment and model is evaluated. This difference is then formulated as an objective function for optimisation based minimisation of the difference. During optimisation the constitutive parameters are iteratively adjusted to minimise the difference with the experimental data. The iFEA procedure used here utilises the experimental data from mechanical testing of the rectangular scaffold (Fig 6). Given the parallel fiber arrangement along the loading direction, and given the uniaxial conditions, the load in each fiber is fully equivalent. Hence, to take advantage or symmetry, the forward model consists of only a single fibre (Fig 7), 10 mm in length and 350 *μ*m in diameter. Tension was applied by prescribing the upward displacement of the top nodes, while also constraining their radial motion, and by fully constraining motion of the bottom nodes. These boundary conditions match the experimental boundary condition (where nodes are constrained due to the fixed bone plugs). Next the simulated tensile forces where exported, and multiplied by 28 to allow for comparison to the experimental data for the full set of 28 fibers.

In total, two optimisations were conducted. First, the elastic properties of the PCL material were optimised. Then, optimised elastic constants were used to optimise viscoelastic properties. The final optimised material constants were used to evaluate the mechanical response of the ligament-scaffold under load from physiological wrist motion. Each iteration of the optimisation involves creating an FEBio input file with the appropriate material parameters, starting FEA, importing and analyzing results, comparing FEA results to the experimental boundary conditions (from mechanical testing) to formulate the objective function, and finally, performing iFEA based optimisation of the objective function using a chosen optimisation algorithm. The inverse parameter identification employed Levenberg-Marquardt [49] based optimisation (the MATLAB *lsqnonlin* implementation).

The constitutive parameters were estimated by minimizing the following objective function vector ***ϕ***:

$$\begin{array}{c} \phi_{i}\left(\ell \right)={\left(F_{i}^{\text{exp}}-F_{i}^{\text{sim}}\right)}^{2} \end{array}$$ (8)

where, $F_{i}^{\text{exp}}$ are the experimental forces and $F_{i}^{\text{sim}}$ the simulated forces, and *i* ∈ [1, *n*] where *n* is the number of experimental data points over time.

The optimisation was deemed to have converged if the sum of squared differences did not vary by more than 0.01.

During the first optimisation step, elastic material behavior only is considered, leading to the material parameter vector *ℓ* = [*c* *m*]. In a second step, the material optimisation includes viscoelastic parameters and employs the parameter vector *ℓ* = [*c* *m* *γ* *τ*].

### Finite element analysis of ligament-scaffold performance

The 3D contours of the scaphoid, lunate, capitate, and radius were derived from segmenting bone regions from X-ray computed tomography (CT) images. These contours were then converted to triangulated surface models, wherein bones were represented as rigid bodies. The ligament-scaffold, in three length permutations (3-, 5-, 7- and 10- mm), was meshed with hexahedral elements, and is deformable, and modelled as a viscoelastic solid using the material parameters identified. The motion of the scaphoid, lunate and capitate bones were fully prescribed by applying the displacements and rotations derived from physiolocal boundary conditions of the wrist. The same resulting physiological boundary conditions were applied to the ends of the ligament-scaffold (Fig 5a, red and black dots) to simulate a rigid BLB interface. This approach enables prescription of physiological motion directly to the ligament-scaffold (or alternatively the bones to which it is attached) based on motion from medical images. The von Mises stress and maximum von Mises stresses were calculated.

### Evaluation of scaffold design

To assess ligament-scaffold performance, candidate surgical installation sites were subject to physiological wrist motion and calculated maximum and 3D von Mises stresses were used as failure criteria. Yield strength of PCL was derived from the average of the experimental force-displacement curve and used as critical stress.

A process to select the optimal configuration of scaffold was implemented, based on FEA numerical evaluation, from the described subset of ligament-scaffold lengths and surgical placements. Based on outcomes of the FEA, the ligament-scaffold portion of BLB scaffold was optimised in terms of its length. The BLB scaffold was then digitally placed at candidate surgical sites. Complying with optimisation and assessing safety of installation, the 3D closest point distance was calculated from the surfaces of the optimised scaffold and bone. Based on the experience of co-author RB (specialist wrist surgeon), we considered proximity less than 2 mm as high risk for bone wall fracture during drilling.

## Results

### Inverse finite element based mechanical properties

**Fig 8:**
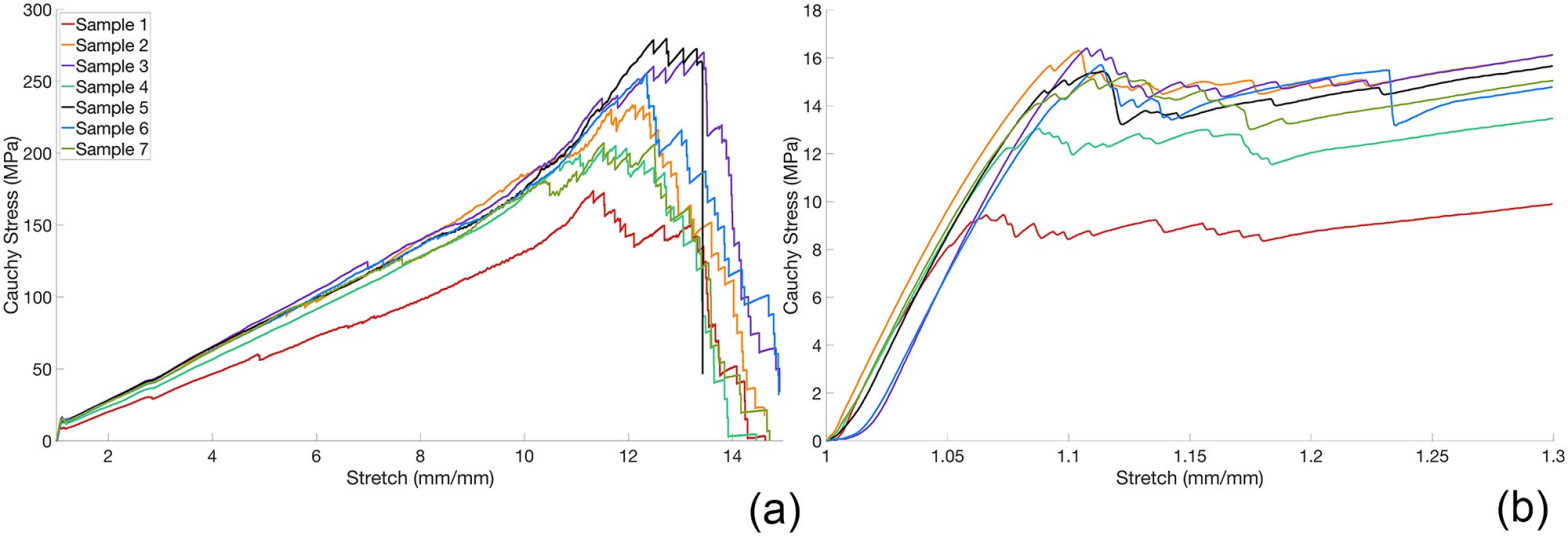
The Cauchy stress-stretch curves. (a) The 3D-printed samples. The zoom in (b) locates the elastic-plastic region from Cauchy stress-stretch curves depicted in (a).

**Fig 9:**
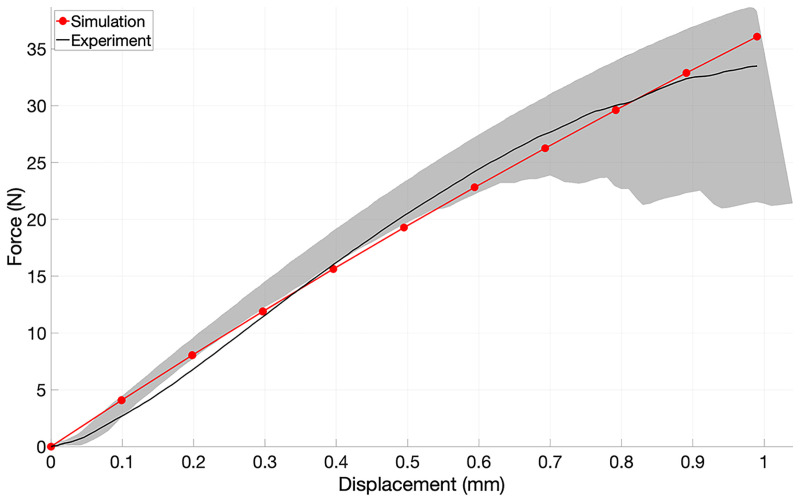
Experimental and simulated force-displacement curves. This is the force-displacement curve using the material constants from the optimisation of the elastic properties. The area between minimum and maximum force-displacement experimental curves are shaded grey.

**Fig 10:**
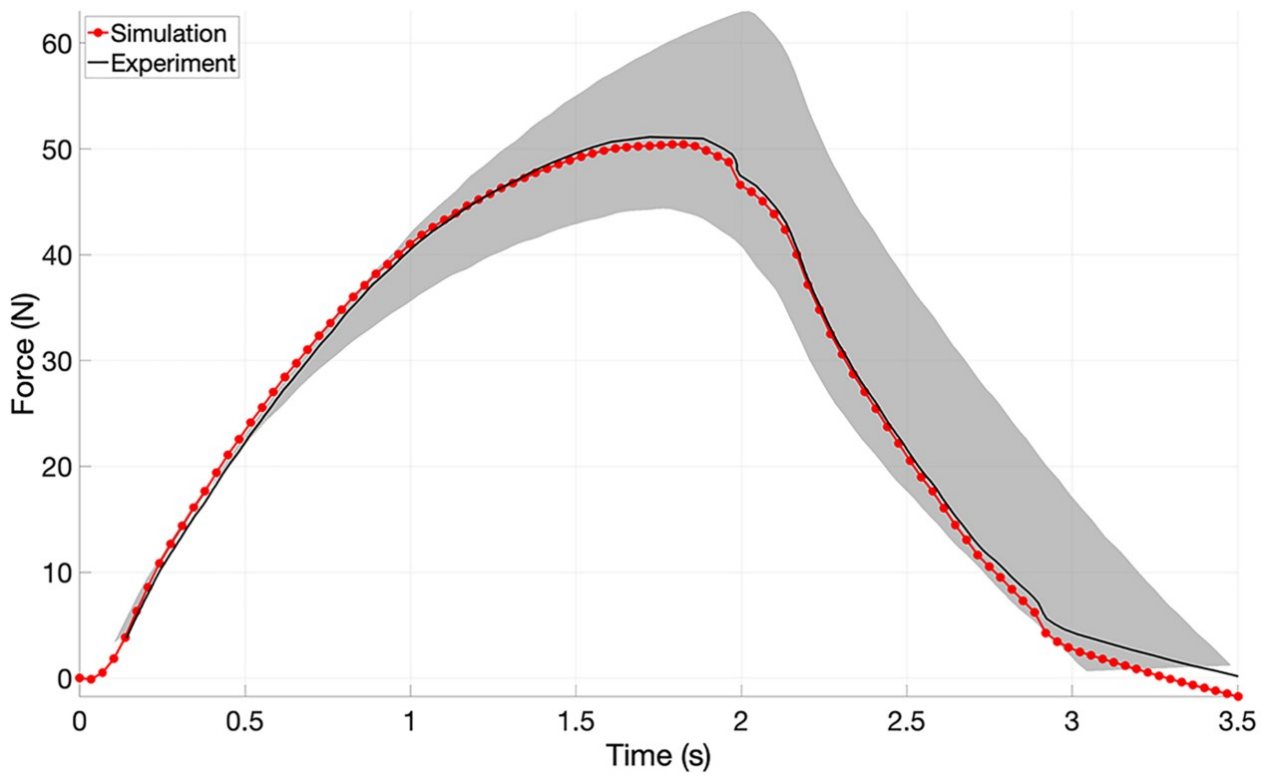
Experimental and simulated force-time curves. This is the force-time curve using the material constants from the optimisation of the viscoelastic properties. The area between minimum and maximum force-time experimental curves are shaded grey.

Cauchy stress-stretch curves ending in fracture and after yield are shown (Fig 8a and 8b, respectively). Reproducibility of tensile curves is good. Average force-displacement and force-time curves are plotted (Figs 9 and 10, respectively). Minimum and maximum experimental curves are shaded gray, showing averages are good representations of experimental data.

Results of material constant optimisation are summarized (Table 1).

**Table 1:** Optimised material parameters for finite element models.

*c* (kPa)	57.05317
*m*	2.0008
*γ* (MPa)	1.23339
*τ* (s)	25.365

### Physiological boundary conditions

**Fig 11:**
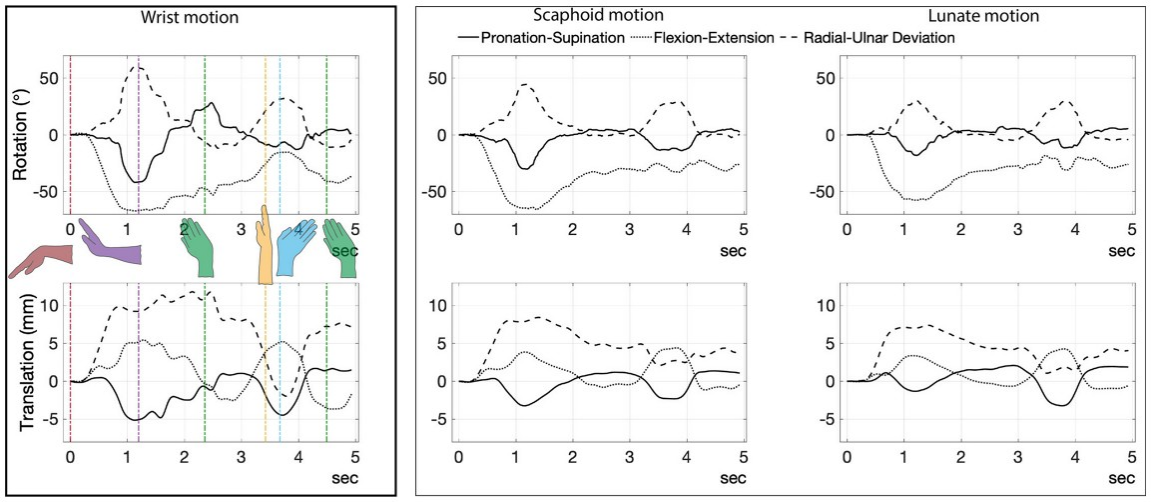
Filtered motion of wrist, scaphoid, and lunate for flexion (first frame) acquired from tri-planar video-radiography [41]. Times 0; 1.11; 2.355; 3.42; 3.67 and 4.485 seconds correspond to flexion (red), extension (purple), radial deviation (green), neutral (yellow), ulnar deviation (cyan) and radial deviation again (green), respectively.

Rotation (^0^) and translation (mm) against time are plotted (Fig 11). These are the magnitude of translations and rotations relative to the wrist’s full flexion position. Wrist flexion, extension, neutral, radial deviation, and ulnar deviation were demarcated using color coding (Fig 11).

### Ligament-scaffold stresses during physiological motion

**Fig 12:**
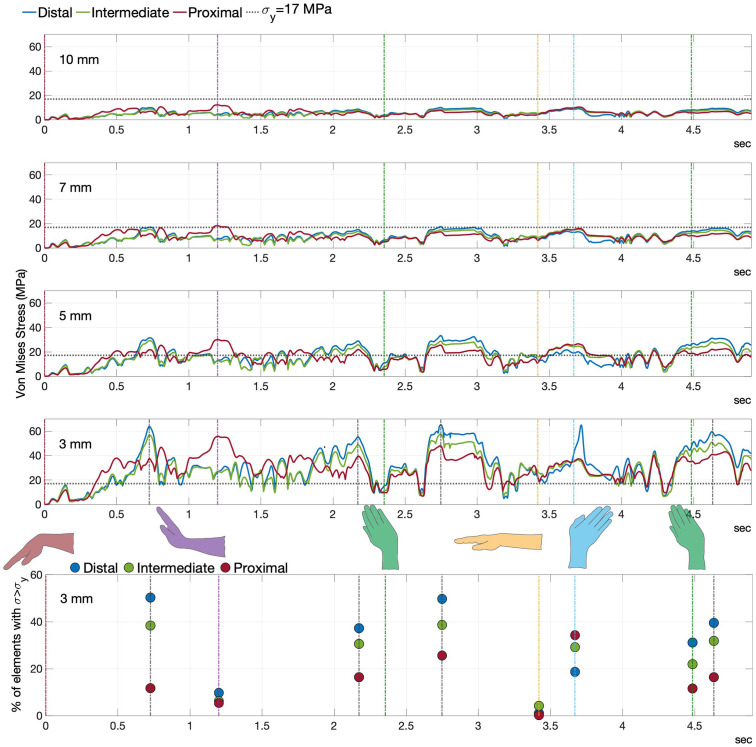
Summary of von Mises stress. The 10-, 7-, 5- and 3-mm ligament-scaffold inserted at distal (Fig 5b), intermediate (Fig 5c), and proximal (Fig 5d) surgical sites. The von Mises stress depicted maximum values of stress distribution in ligament-scaffold. The percentage of mesh elements exceeding critical stress (*σ*_*y*_ = 17 MPa) for 3 mm ligament-scaffold length was calculated at times 0; 1.11; 2.355; 3.42; 3.67 and 4.485 seconds, which correspond to flexion (red), extension (purple), radial deviation (green), neutral (yellow), ulnar deviation (cyan) and radial deviation (green), respectively.

The maximum von Mises stresses from each of the three candidate surgical installation sites and their associated ligament-scaffold lengths are plotted (Fig 12). Critical stress (*σ*_*y*_) is 17 MPa (Fig 8b). There is no obvious effect of surgical installaiton site on stress in the ligament-scaffold (Fig 12). The percentage of mesh elements exceeding critical stress was calculate at a selected time for a representative ligament-scaffold (i.e., 3 mm length) (Fig 12). The ligament-scaffold with 3 mm length shows highest stress profile among different lengths assessed.

The lowest and highest stresses consistently developed when the ligament-scaffold was placed at proximal and distal sites, respectively, and independent of ligament-scaffold length. Only when wrist was at full extension, highest stress developed when ligament-scaffold installed at proximal rather than distal site. The stress in the ligament-scaffold at the intermediate position interposes between those developed from proximal and distal sites.

The percentage of mesh elements with ligament-scaffold with stress above critical value demonstrated lower risk of failure at the proximal site (5 to 30% of mesh elements) and higher risk at distal site (10 to 50% of mesh elements). The ligament-scaffold placed at the intermediate site demonstrated an percentage of elements above critical stress (from 5 to 20%).

Maximum stress in 7- and 10- mm ligament-scaffolds was always beneath critical stress. In contrast, the 3- and 5- mm lengths occasioned maximum stress above critical stress.

**Fig 13:**
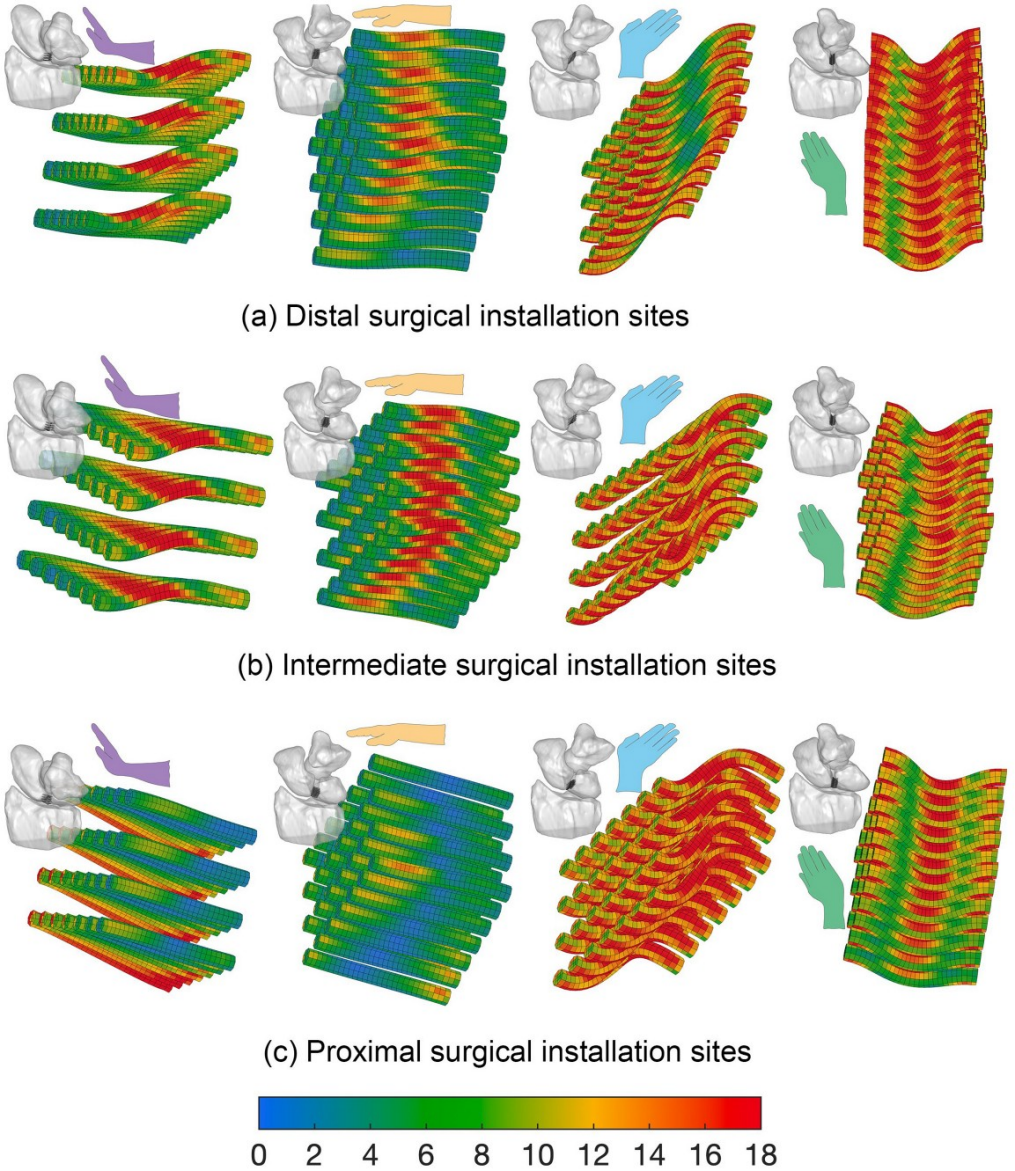
Von Mises stress distribution on the 3 mm ligament-scaffold at three surgical installation sites. (a) The distal (Fig 5b); (b) intermediate (Fig 5c); (c) proximal (Fig 5d) in the dorsal view of the wrist being in positions of extension, neutral, ulnar deviation, and radial deviation. The von Mises stress scale bar is from 1 to 18 MPa.

**Fig 14:**
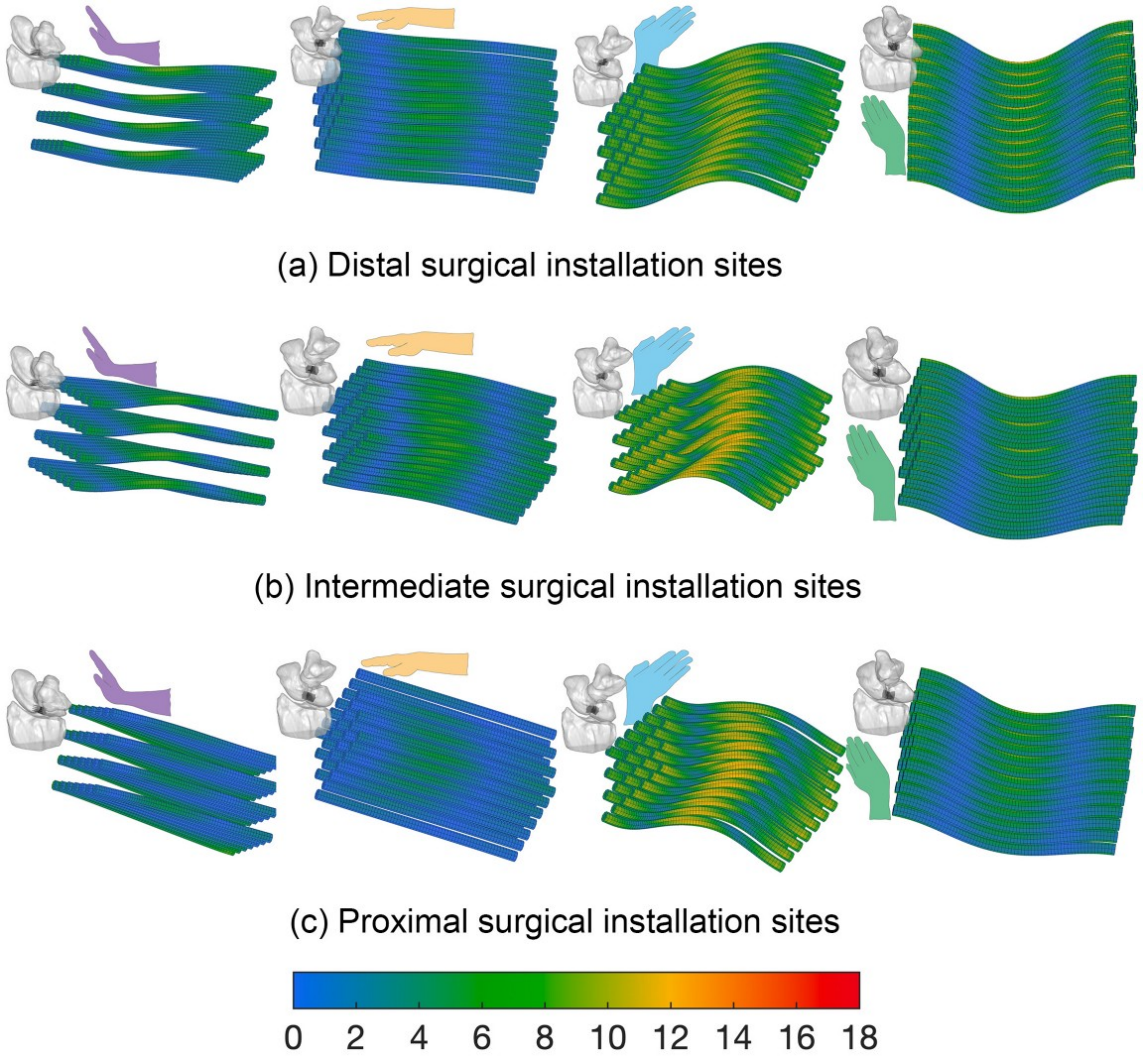
Von Mises stress distribution on the 7 mm ligament-scaffold at three surgical installation sites. (a) The distal (Fig 5b); (b) intermediate (Fig 5c); (c) proximal (Fig 5d) in the dorsal view of the wrist being in positions of extension, neutral, ulnar deviation, and radial deviation. The von Mises stress scale bar is from 1 to 18 MPa.

The 3D von Mises stress distribution patterns for 3- and 7- mm ligament-scaffold lengths and three candidate surgical installation sites are presented for 3 mm (Fig 13) and 7 mm (Fig 14). The 7 mm ligament-scaffold length had a more consistent stress distribution pattern compared to the 3 mm length variant. For the 7 mm length, and across the candidate installation sites and wrist activities, high levels of stress developed in both middle and sides of the ligament-scaffold. For 3 mm length, overall stress development was scattered depending on installation site and wrist activity.

**Fig 15:**
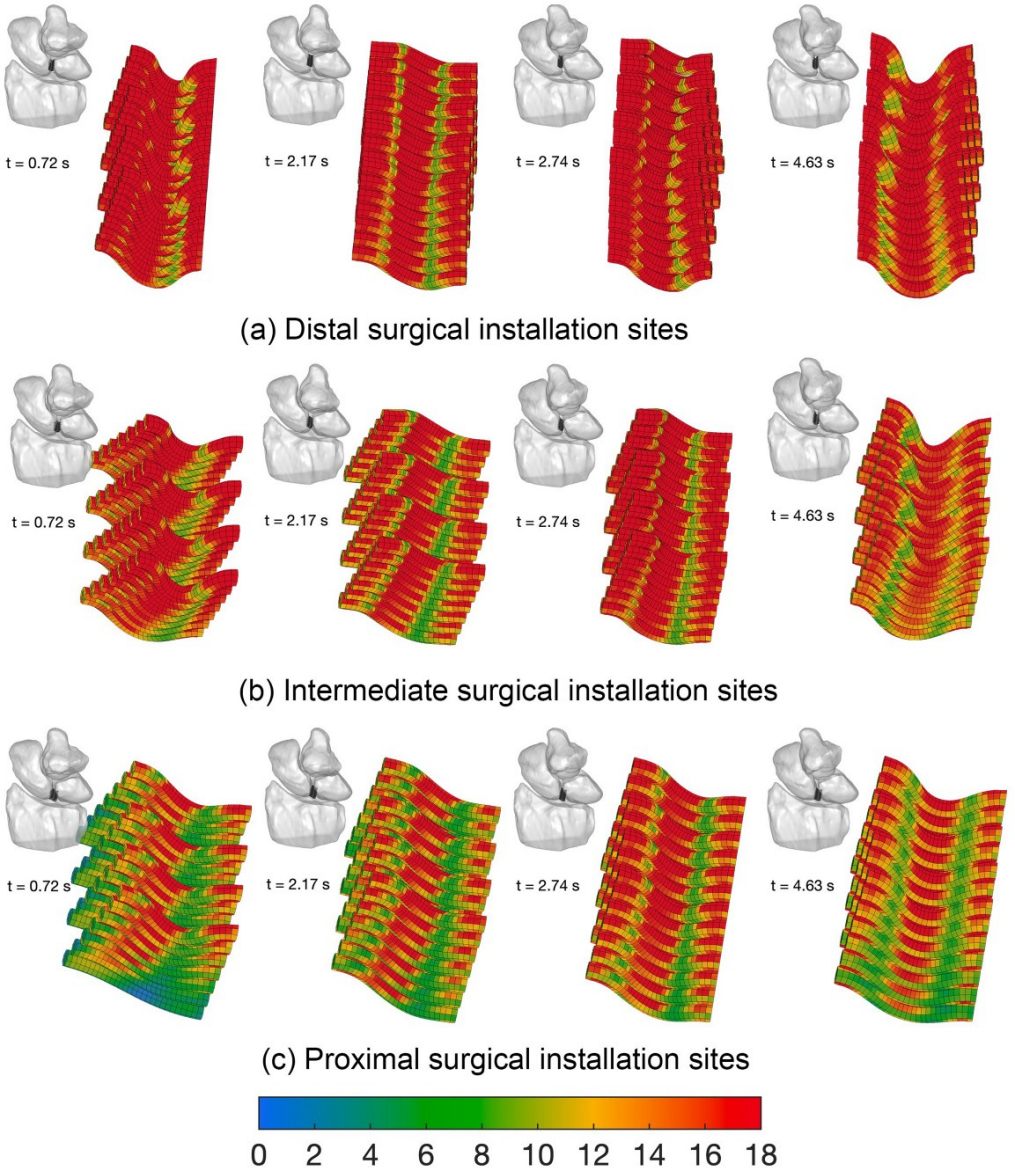
Von Mises stress distribution on the 3 mm ligament-scaffold at three installation surgical sites. Chosen time corresponds to maximum peaks in von Mises stress (Fig 13). (a) The distal (Fig 5b); (b) intermediate (Fig 5c); (c) proximal (Fig 5d) in the dorsal view of the wrist.

The von Mises stress patterns on the 3 mm scaffold (Fig 13) corresponded to certain wrist motions (Fig 15). Specifically, stress peaks develop during radial deviation, as the wrist transitioned between maximal wrist positions.

### Design evaluation

**Fig 16:**
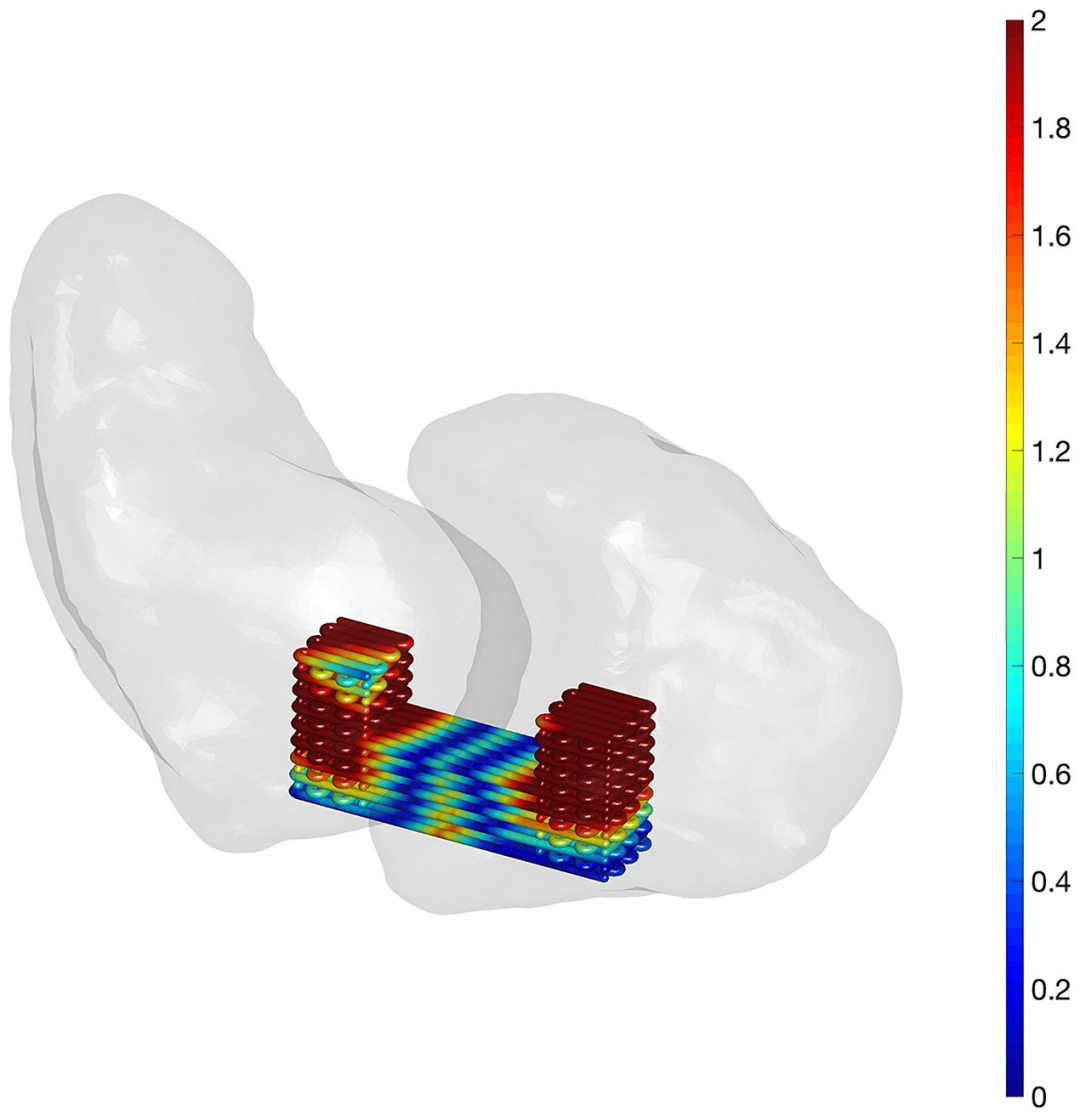
Closest point distance (mm) from an optimised scaffold to scaphoid and lunate. The length of the ligament-scaffold is 7 mm. The scaffold is placed at the proximal site according to Fig 5d. The scale bar is in the range from 0 to 2 mm.

The 3D closest point distance was calculated from optimised scaffold’s surface to bone surfaces (Fig 16). The distance less than 2 mm is coded in blue and presented on the scaphoid side in the scaffold.

## Discussion

Reconstruction of the scapholunate interosseous ligament remains a challenge for hand surgeons with various procedures proposed but no reliable solution. An engineered scaffold providing mechanical support to the dissociated scaphoid and lunate bones while also encouraging ligament formation has shown promising results in animal models. However, use in the human wrist requires precision design, manufacture, and installation. The aim of this paper was to develop a computational framework to design a personalised scaffold and further, assist the surgeon plan the optimal site for scaffold installation based on *in silico* modelling of the ligament-scaffolds performance in the wrist.

We studied the 3D-printed PCL multiphasic scaffold design previously developed by our colleagues for animal models (Fig 2, [27]). The goal of reconstruction with a printed multiphasic scaffold is to implant the bone plugs into drilled holes in the scaphoid and lunate with the longitudinal fibers aligned on the dorsal surface of the scapholunate articulation. Over time, bone plugs ideally fully incorporate into scaphoid and lunate while the ligament-scaffold bridge is replaced with collagen aligned to form a novel biologically restored SLIL. The scaffold is designed to enable bone growth into its pores (i.e., voids) within the terminal bone plugs, which also provide rigid fixation required for bone in-growth into an implant. We assumed the ligament-scaffold will bear the major loads produced by wrist motion. Therefore, it is important the stress developed in the ligament-scaffold does not lead to failure. Consequently, FEA models were created to evaluate maximum and 3D stress development by varying ligament-scaffold lengths and positioning at feasible surgical sites, all subjected to physiologic wrist motion. The power of studied FEA was the incorporation of subject-specific anatomy, coupled with dynamic motion data derived from tri-planar videoradiography [41], and optimised material parameters of PCL material model. Many previous studies used two X-ray sources (i.e., biplanar videoradiography), but in this study three X-ray sources were used to achieve a higher accuracy in tracking the motion of carpal bones, specifically, capitate, scaphoid, and lunate used for FEA simulations.

FEA models of the wrist have been previously developed for various purposes to study load transmission [50–54], behavior of a bone after fracture fixation [55–60], vibration transmission [61, 62], carpal bone replacement [63] and fingertip-object interaction [64, 65]. One of the strategies that has been used for studying wrist motion in 3D FEA is load transfer to metacarpal bones at single or multiple points while reproducing critical wrist postures in a quasi-static manor. In this approach, a model of the entire wrist is constructed, including (e.g. [52–54]) or excluding (e.g. [66]) their articulations. Another strategy has been focused on quasi-static 2D (e.g. [50, 57, 63]) or 3D (e.g. [51, 55, 56, 58]) FEA of particular joint(s) with force or displacement control modes mimicking particular physiological states.

Conversely, the 3D FEA models of SL joints developed in this study feature displacement control to capture detailed wrist kinematics with a high temporal resolution. This approach enables accurate analysis across a multitude of time points during the motion cycle.

Previously published FEA work of the wrist is based on quasi-static loading, corresponding to a certain point in the time of wrist motion. Our FEA model includes continuous kinematic motion of the full wrist motions. This gives an opportunity to evaluate stress distributions over time during physiological wrist motion—an achievement not previously reported in the literature. Furthermore, published FEA studies of 3D-printed scaffolds [32, 59] cover only bone plug of current construct (see Fig 2). Due to novelty of current design and application, no one so far has evaluated the ligament-scaffold performance which plays an important role bearing the major loads from wrist motion.

Calculated maximum and 3D von Mises stress were use as a failure criterion. The proximal surgical site generated von Mises stress below critical levels using 7 to 10 mm ligament-scaffolds, whereas 3- and 5- mm lengths will fail is subjected to physiologic wrist motion. Under physiological wrist motion, the ligament-scaffold stresses appear to be more influenced by ligament-scaffold length and wrist posture than surgical installation sites. The 7 mm length demonstrates consistency in stress distribution in the ligament-scaffold whereas stress is scattered in the 3 mm length.

Our numerical results show that stresses were larger in shorter specimens and, therefore, generated larger strains for a given displacement. The mechanics of the native SLIL have not received as much research focus as hand-wrist tendon, but tendons and ligaments possess similar ultrastructure and physiology, as well as fulfilling similar functions in particular cases [67]. Uniaxial tensile experiments on rat-tail tendon [68, 69] demonstrate larger total strains at short compared to long specimen lengths. Stresses, on the other hand, were insensitive to specimen length. Further experimental studies suggested this strain sensitivity might be a technical artifact (i.e., “end-effect”), where extra unseen sample length within the grips of a mechanical testing machine contributes dramatically to overall strain measurements in short specimens, whereas the response of longer samples more closely reflects true material properties [70, 71]. Therefore, acquiring both simulation and experimental results for validation is necessary. Experiments on cadaver is our target for future studies.

The wrist in a flexed posture was used as the reference position for studied FEA modelling. Therefore, scaffold stress at this position was low. In all simulations the different scaffold lengths, the maximum von Mises stress were substantially greater at the position of maximum wrist extension and the transition motions: from flexion to extension; from extension to radial deviation; from radial deviation to neutral; from neutral to ulnar deviation; from ulnar to radial deviation. The transition motions show pics in stress. These observations concur with a previous study [3] and suggest patients should limit their wrist extension and transition motions in the early period after surgery involving SLIL reconstruction.

Using anatomical models created from CT, we performed a semi-quantitative analysis of risks for bone fracture at the candidate surgical installation sites. In this approach, the closest distance between scaffold bone plugs and scaphoid and lunate out surfaces was quantified. Based on the extensive experience of co-author RB (specialist wrist surgeon), the proximity less than 2 mm was considered as high risk for bone wall fracture. Moreover, there is a concern that the height of bone plug of 3 mm or less will not provide a secure bone-plug interface and resulting micromotion will have a negative impact on bone regeneration. The proximal site for SLIL reconstruction (i.e., scaffold installation) offers more bone volume for drilling and subsequent plug insertion, however, the location is proximal to the native SLIL footprint and this may impair regeneration and potentially intrude on other tissues. In contrast, the distal site offers comparatively limited bone volume to accommodate the current scaffold design, but perhaps ideal mechanical and functional properties. A balance between installation safety and mechanical function is suggested.

The scaffold design (Fig 2) warrants further optimisation to enhance bone plug fixation into the proximal position to be safely placed proximally in the dorsal view of the wrist.

### Limitations

The major limitation of the current work is the motion data used to drive the FEA simulations were collected from a single cadaveric specimen. At present, acquiring both motion and force data from the small wrist bones, such as the scaphoid and lunate, during dynamic activities is exceedingly difficult using 3D optical motion capture or biplanar videoradiography. This difficulty is primarily due to the small size of these bones and how they move relative to the other bones and tissues, often occluding line of sight or causing collision between stand-off marker frames. Although wrist motion has previously be calculated from other types of medical imaging, such as those found on the Carpal Database (wrist anatomy and kinematics, created using static CT wrist positions, [72]), kinematics generated using the Carpal Database are not comparable to tri-planar videoradiography due to the different wrists studied and methods for computing kinematics. The Carpal Database uses a mathematical model that describes wrist motion based on quadratic surface models generated based on static bone poses. The current data were collected using tri-planar videoradiography, which we contend is the most appropriate and comprehensive data collected on wrist motion. Therefore, we are confident with the results computed tri-planar videoradiography and FEA simulations.

Another limitation of this study is the challenge of optimising the ligament-scaffold fibre number and diameter. This is the outside of our scope. We anticipate changing fibre number will constrain bone motions, which in turn will wrist function.

Finally, finite element models were not directly validated against measures of strain in vivo or in situ. Ongoing experiments in our laboratory will explore whether robotically actuated cadaveric wrists, when implanted with the scaffold, can provide insight into scaffold mechanics under physiological motion and force boundary conditions. Although the developed models can be used to provide insight into scaffold mechanics when experimental data are not available, the validity of our predictive simulations will only be as good as the assumptions and approximations used.

## Conclusion

In conclusion, it is advisable to use longer scaffolds whenever possible to reduce the stresses and strains and their variability during physiological wrist motion. The choice of surgical installation site can be very sensitive to the patient’s bone anatomy and natural wrist motion. Therefore, the choice of surgical installation site should be evaluated using individual wrist motion and bone anatomy of the patient. It is recommended to avoid full extension of the wrist and transition motions between wrist critical positions avoiding development of high stress and strain in scaffold. Future mechanical evaluation is needed to experimentally confirm these findings.
